# The Chemotactic Defect in Wiskott-Aldrich Syndrome Macrophages Is Due to the Reduced Persistence of Directional Protrusions

**DOI:** 10.1371/journal.pone.0030033

**Published:** 2012-01-18

**Authors:** Dan Ishihara, Athanassios Dovas, Haein Park, Beth M. Isaac, Dianne Cox

**Affiliations:** 1 Department of Anatomy and Structural Biology, Albert Einstein College of Medicine, Bronx, New York, United States of America; 2 Department of Developmental and Molecular Biology, Albert Einstein College of Medicine, Bronx, New York, United States of America; Université de Genève, Switzerland

## Abstract

Wiskott-Aldrich syndrome protein (WASp) is an actin nucleation promoting factor that is required for macrophages to directionally migrate towards various chemoattractants. The chemotaxis defect of WASp-deficient cells and its activation by Cdc42 *in vivo* suggest that WASp plays a role in directional sensing, however, its precise role in macrophage chemotaxis is still unclear. Using shRNA-mediated downregulation of WASp in the murine monocyte/macrophage cell line RAW/LR5 (shWASp), we found that WASp was responsible for the initial wave of actin polymerization in response to global stimulation with CSF-1, which in *Dictyostelium discoideum* amoebae and carcinoma cells has been correlated with the ability to migrate towards chemoattractants. Real-time monitoring of shWASp cells, as well as WASp^−/−^ bone marrow-derived macrophages (BMMs), in response to a CSF-1 gradient revealed that the protrusions from WASp-deficient cells were directional, showing intact directional sensing. However, the protrusions from WASp-deficient cells demonstrated reduced persistence compared to their respective control shRNA and wild-type cells. Further examination showed that tyrosine phosphorylation of WASp was required for both the first wave of actin polymerization following global CSF-1 stimulation and proper directional responses towards CSF-1. Importantly, the PI3K, Rac1 and WAVE2 proteins were incorporated normally in CSF-1 – elicited protrusions in the absence of WASp, suggesting that membrane protrusion driven by the WAVE2 complex signaling is intact. Collectively, these results suggest that WASp and its phosphorylation play critical roles in coordinating the actin cytoskeleton rearrangements necessary for the persistence of protrusions required for directional migration of macrophages towards CSF-1.

## Introduction

Monocytes and macrophages circulate throughout the organism and their ability to be recruited to various sites is crucial for their function as important mediators of innate and adaptive immunity. Cellular migration can be mechanistically described as a sequence of events that repeats until the cell reaches its destination. The initial event in cell migration is polarization and extension of protrusions in the direction of migration [1]. These protrusions persist and are stabilized through the formation of new adhesions to the underlying substratum. The cell body then moves forward, propelled by actin-myosin mediated contraction. Finally, the tail of the cell detaches from the substratum and retracts [2]. A popular model of chemotaxis hypothesizes that, while chemotactic receptors are evenly distributed over the cell surface, the cells utilize receptor-mediated signaling network as a molecular “compass” to sense small differences of chemoattractant outside of the cell leading to directional migration [3].

In response to migratory stimuli, macrophages polarize and extend F-actin rich protrusions in the direction of the chemotactic gradient [4], [5]. The protrusive force required for the formation and extension of these structures is controlled by the reorganization of the actin cytoskeleton [6]. Wiskott-Aldrich Syndrome protein (WASp), along with neuronal WASP (N-WASP) and WASp family Verproline-homologous protein (WAVE)1, 2 and 3, are members of a family of scaffold proteins that links signals from the cell-surface to the actin cytoskeleton [7]. WASp family proteins promote *de novo* Arp2/3 complex dependent actin polymerization [8]. Expression of WASp is restricted to non-erythroid hematopoietic cells [9] and the essential role of actin cytoskeleton dependent processes in leukocytes is exemplified by the cytoskeletal abnormalities of hematopoietic cells from WAS patients (reviewed in [10]).

Macrophage colony stimulating factor-1 (CSF-1) is a potent chemoattractant and a pleiotrophic growth factor that stimulates cell survival, proliferation and differentiation of myeloid-derived cells (reviewed in [11], [12]). CSF-1 binding to the CSF-1 receptor (CSF-1R) tyrosine kinase initiates a number of signaling cascades, including PI 3-kinase (PI3K) and Rho family GTPases, leading to actin reorganization and migration (reviewed in [13]). Rac and WAVE2 form a complex of proteins that mediate CSF-1-induced actin polymerization and macrophage motility [14], [15]. Interestingly, Allen and colleagues found dominant negative Cdc42 mutant macrophages were still able to migrate but unable to polarize or directionally migrate towards CSF-1 [4]. The observed chemotaxis defect of this mutant macrophage is strikingly similar to that of macrophages from WAS patients, which exhibit enhanced motility in response to CSF-1 but are unable to migrate directionally [16]. PI3K and Cdc42 mediate WASp activation downstream of global CSF-1stimulation *in vivo* [17]. Moreover, we have demonstrated that the Src-dependent phosphorylation of WASp on Y291 is important for macrophage chemotaxis towards CSF-1 [18]. Collectively, these studies suggest that Cdc42 and tyrosine phosphorylation regulate WASp activity during directional migration. However, the precise role WASp plays in actin cytoskeleton during macrophage chemotaxis has not been delineated.

Chemoattractant-mediated biphasic responses of the actin cytoskeleton are observed in motile cells, such as *Dictyostylium* amoebae, neutrophils, and carcinoma cells, where an initial spike in actin polymerization that correlates with directional sensing is followed by secondary responses over the next several minutes that are associated with motility [3]. In examining the sequential rearrangements of the actin cytoskeleton in macrophages, Diakonova and colleagues reported that macrophages respond to CSF-1 with a transient but broad increase in F-actin content within a minute and slowly decrease over the course of five minutes [19]. Our previous studies have shown that WASp activation occurs rapidly in response to CSF-1 [17], coinciding with the first F-actin peak. Hence, we hypothesized that WASp could be playing a role in the initial transient increase in F-actin content of macrophages in response to CSF-1 that is required for directional migration in macrophages.

## Materials and Methods

### Mice

All procedures involving mice were conducted in accordance with National Institutes of Health regulations concerning the use and care of experimental animals. The Albert Einstein College of Medicine animal use committee approved the use of mice in this study (Approval ID 20110412). Commercially available 129/svJ wild-type and WASp^−/−^ mice [20] were purchased from The Jackson Laboratory (Bar Harbor, ME).

### Cells, transfections, plasmids

All cells were maintained at 37°C in a 5% CO_2_ incubator. Murine RAW/LR5 monocyte/macrophages [21] with reduced WASp expression by short hairpin RNA (shWASp) [18], shWASP cells stably expressing a phospho-deficient WASp (Y291F) and shControl cells expressing non-targeting shRNA were described in [18] and were grown in RPMI medium (Mediatech Inc, Manassas, VA) containing 10% newborn calf serum (Cambrex, Walkersville, MD), 100 U/ml penicillin and 100 ug/ml streptomycin (Sigma, Saint Louis, MO). Murine bone marrow-derived macrophages (BMM) were isolated and prepared according to a previously published protocol [22], and grown in alpha-MEM media (Invitrogen, Carlsbad, CA) with 15% FBS (Sigma, St. Louis, MO), 36 ng/ml recombinant human CSF-1 (Chiron, Emeryville, CA), and 100 U/ml penicillin and 100 ug/ml streptomycin.

### Immunofluorescence microscopy

RAW 264.7 derived cell lines were plated on 12 mm glass coverslips and serum-starved for at least 3 hours in RPMI prior to stimulation with CSF-1 at either 37°C or 22°C, followed by fixation and staining according to [18]. Wiskostatin was added at 5 µM for 1 hour prior to CSF-1 stimulation. F-actin was visualized by staining with Alexa 568-phalloidin (Invitrogen, Carlsbad, CA) images were captured below the saturation level using the 20× air/1.40 phase1 objective of an Olympus IX71 microscope coupled to a Sensicam cooled CCD camera. Ruffling index was determined as previously described [21]. Quantitation of the F-actin content of single cells was achieved by measuring the total pixel fluorescence intensity of single cells using ImageJ (http://rsb.info.nih.gov/ij/) [14]. At least 30 cells were analyzed per experiment for each condition and cell type.

The kinetics of total F-actin content in response to CSF-1 using a plate reader were quantified according to [23]. Fixed cells were incubated with saturating concentrations of rhodamine-phalloidin and YO-PRO-1 (both from Molecular Probes) to stain F-actin and nucleic acids, respectively. Fluorescence intensities of rhodamine (excitation wavelength: 545 nm, emission wavelength: 590 nm) and YO-PRO-1 (excitation wavelength: 485 nm, emission wavelength: 520 nm) were measured using a Polarstar Optima plate reader (BMG Labtech, Cary, NC) and the normalized F-actin cellular content (calculated as the ratio of rhodamine to YO-PRO-1 fluorescence) was expressed as the percentage of increase in response to CSF-1 compared to the unstimulated condition.

### Directional CSF-1 stimulation

The micropipette assay was performed based on [24] using a Femtojet® manipulator Patchman NP 2 (Eppendorf-Brinkman Instruments) and a pump (model Femtojet® Eppendorf) to control the position of the micropipette and the pressure required for CSF-1 flow. BMMs were CSF-1 deprived overnight and RAW/LR5-derived cell lines were serum starved in RPMI media overnight. A micropipette filled with 10 nM CSF-1 under a pressure of 17 hPa exerted to induce flow, was introduced to induce the formation of protrusion. Time-lapse images were taken every 5 seconds for the duration of 6 minutes, where the cells were imaged for one minute prior to the insertion of the micropipette to obtain a base-line level of protrusion using a 20× air/1.40 phase1 objective of an Olympus IX71 microscope. Analysis of captured images was performed using ImageJ. Greater than 90% of the cells in each genotype and cell line responded to the introduced micropipet. Measurements and protrusion rates were performed as described previously [25]. Directional protrusion percentage was calculated by dividing the number of directional protrusions over the total number of protrusions recorded. Chemotactic index was measured as the cosine θ after stimulation with a CSF-1 containing pipette, as described [26]. The angle θ is defined by two reference lines: a line going through the first and the last centroid and a line going through the first centroid and the position of the pipette tip. Retraction percentage was calculated by dividing the number of protrusions that completely retracted to the border of plasma membrane over the total number of protrusions recorded. At least 30 protrusions from seven independent experiments were analyzed for each cell type.

### Pseudopod isolation and western blotting

WT or WASP^−/−^ BMMs (1×10^6^) were deprived of CSF-1 for 14 hours before being loaded onto 3-µm pore size inserts, with media containing CSF-1 in the lower chamber, and incubated at 37°C for 30 min. Pseudopods from the lower surface of the inserts and total cell lysates were obtained by lysis in ice-cold buffer A (25 mM Tris, 137 mM NaCl, 1% NP-40, 2 mM EDTA, 1 mM orthovanadate, 1 mM benzamidine, 10 µg/ml aprotinin, 10 µg/ml leupeptin, pH 7.4). Lysates were resolved by SDS-PAGE, proteins were transferred onto PVDF membranes (Immobilon-P Millipore, Billerica, MA) followed by blocking and incubation in primary antibodies overnight at 4°C. Membranes were blotted with antibodies against β-actin (clone AC-15, Sigma, St. Louis, MO), CD14 (clone rmC5-3; BD Pharmingen), p85 (4292; Cell Signaling Technology), Rac (C14), WASp (H250; Santa Cruz Biotechnology, Santa Cruz, CA), and WAVE2 (Yamazaki et al., 2003). Membranes were then washed and incubated with horseradish peroxidase conjugated secondary antibodies against rat, mouse or rabbit IgG (Jackson Immuno Research, West Grove, PA). Signals were visualized using the SuperSignal West Pico Chemiluminescent Substrate from Pierce (Rockford, IL). Images were acquired using a Kodak Image Station 440 and quantified using Kodak 1D Image Analysis Software. Relative protein levels in pseudopods were quantified by densitometry and normalized to CD14 protein levels. Reported values are the average of 3 independent experiments.

### Data analysis

Results were considered statistically different when analysis using a Student t-test resulted in differences between two means with a p value of less than 0.05. Error bars signify standard error of the mean.

## Results

### WASp is required for the first wave of F-actin increase in response to CSF-1

**Figure 1:**
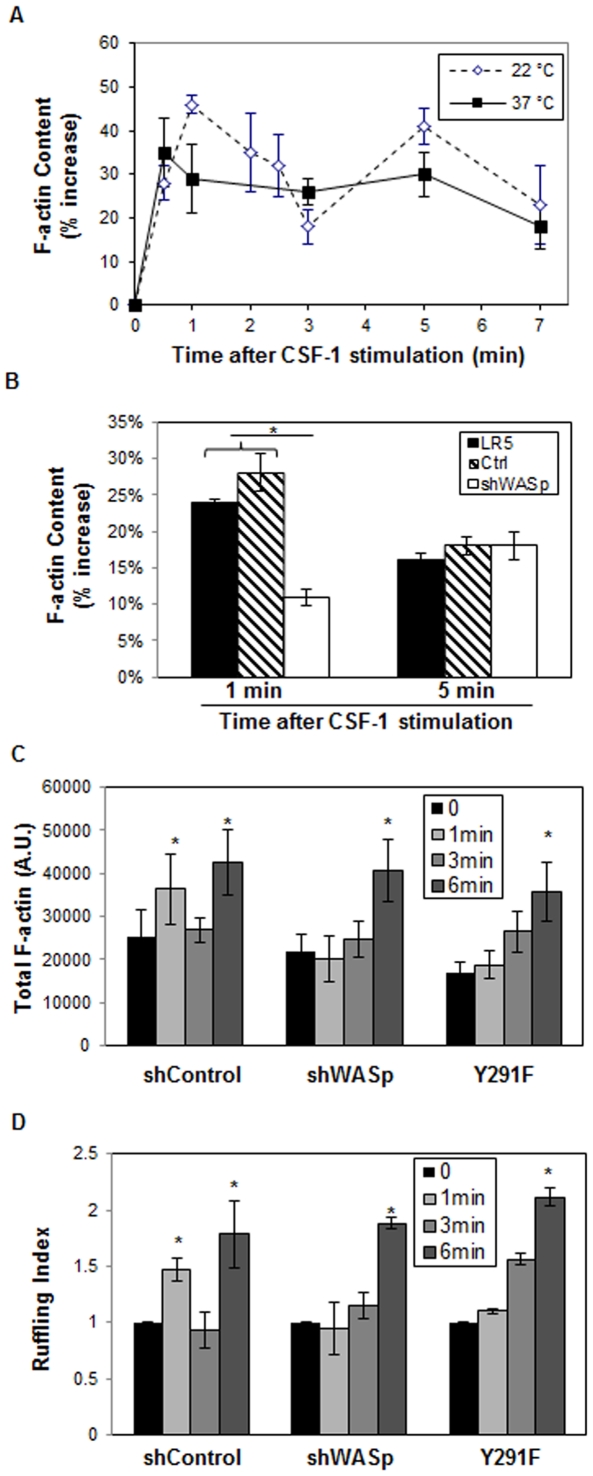
WASp is required for the first wave of actin assembly in response to CSF-1. (**A**) F-actin content of RAW/LR5 cells in response to CSF-1 stimulation at 37°C or 22°C was quantitatively measured and normalized to the cell number, as described in Material and Methods. (**B**) F-actin content of RAW/LR5 (LR5), shControl (Ctrl) and shWASp cells in response to CSF-1 stimulation at 22°C was measured as in (A). n = 3–4 experiments per condition. (**C**) Single cell analysis of F-actin content of shControl, shWASp and Y291F cells in response to CSF-1 stimulation at 22°C. n = 3 experiments, minimum 30 cells were analyzed per condition. (**D**) Ruffling index of shControl, shWASp and Y291F cells in response to CSF-1 stimulation at 22°C. n = 3 experiments, minimum 30 cells were analyzed per condition. Error bars represent SEM. * p<0.05 compared to unstimulated condition.

CSF-1R stimulation results in a transient and broad increase of newly synthesized F-actin in macrophages [19]. On the other hand, EGF stimulation of its receptor, which also belongs to a family of receptor tyrosine kinases, has been shown to induce two transient increases F-actin of motile carcinoma cells [27]. Interestingly, the latter study measured the F-actin content at room temperature (22°C) and other studies using *Dictyostelium discoideum* have found enhanced temporal resolution of actin nucleation following equilibration at reduced temperature [28], indicating that lowering the temperature may slow down cellular processes to sufficient degree to resolve the two transient waves of F-actin content. To explore whether the dynamics of actin assembly in macrophages in response to CSF-1 consists of two transient waves of activities, a time-course of the F-actin content of murine RAW/LR5 cells [21] in response to CSF-1 was examined. In agreement with a previous studied performed on BMMs [19], CSF-1 stimulation of RAW/LR5 macrophages at 37°C resulted in a broad increase in the F-actin content within the first five to six minutes. In order to test whether this increase in F-actin content could be further resolved, we repeated the CSF-1 upshift assay at room temperature (22°C). CSF-1 stimulation of these cells at lower temperature revealed that two waves of actin polymerization did indeed exist in macrophages, where the first wave peaked between 30 s to 1 minute and the second between 4 to 6 minutes, consistent with reports from other motile cells (Figure 1A).

Since the observed kinetics of the first wave of increase in F-actin content coincided with our previously published kinetics of WASp activation status in response to CSF-1 [17], we repeated the CSF-1 upshift experiment using shWASp RAW/LR5 cells, which have significantly reduced WASp expression and have been previously shown to recapitulate the chemotactic defects observed in macrophages isolated from patients with Wiskott-Aldrich syndrome [18]. Examination of the F-actin content of shWASp cells in response to CSF-1 stimulation at room temperature revealed that the first wave of actin polymerization was diminished while the parental or shControl cells showed a transient increase. However, the shWASp cells showed intact second wave of actin polymerization, comparable to that of shControl cells (Figure 1B). To confirm this result, F-actin contents of CSF-1 stimulated shControl and shWASp cells were examined at room temperature at the single cell level. Analysis of shWASp cells showed diminished first wave but intact second wave of F-actin content compared to the shControl cells (Figure 1C), in agreement with the results obtained using a plate reader and suggested that WASp only played a role in the first wave of F-actin increase following CSF-1 stimulation.

Since tyrosine phosphorylation of WASp has been suggested to regulate WASp activity and is required for macrophage migration [18], [29], the role of WASp phosphorylation in the early responses of F-actin assembly in macrophages was examined. Stable shWASp cells rescued with a phospho-defective WASp point mutant (Y291F) did not display an increase in F-actin content after 1 minute of CSF-1 stimulation while a significant increase was found at 5 minutes of stimulation (Figure 1C). These results suggested phosphorylation of WASp played a specific role in the initial wave of CSF-1 induced F-actin increase. Furthermore, the observation that the second transient wave of actin assembly is intact in WASp-deficient or phospho-defective mutant cells, is consistent with previous reports showing that WAVE2 [15] regulates the formation of F-actin rich membrane ruffles that are prominent after five minutes of CSF-1 stimulation. Hence, we proceeded to examine the membrane ruffles of control and shWASp cells. In agreement with the result of F-actin kinetics, analysis of membrane ruffles showed shWASp cells did not respond to CSF-1 during the first wave of actin activity but ruffled to comparable level of shControl cells during the second wave of actin polymerization (Figure 1D). Moreover, examination of ruffling responses of Y291F mutants also showed these cells ruffled at the later time point but not after 1 minute of CSF-1 stimulation, suggesting WASp and its phosphorylation status only plays a role in the first wave of actin assembly and its absence does not affect the second wave of activity. To further confirm the role of WASp in the first wave of actin assembly, we treated the control cells with the WASp inhibitor Wiskostatin prior to stimulation with CSF-1. In agreement with the results obtained using shWASp cells, Wiskostatin suppressed the increase in F-actin content in response to CSF-1, unlike DMSO treated controls (data not shown).

### WASp is required for the persistence of protrusions and not directional sensing

**Figure 2:**
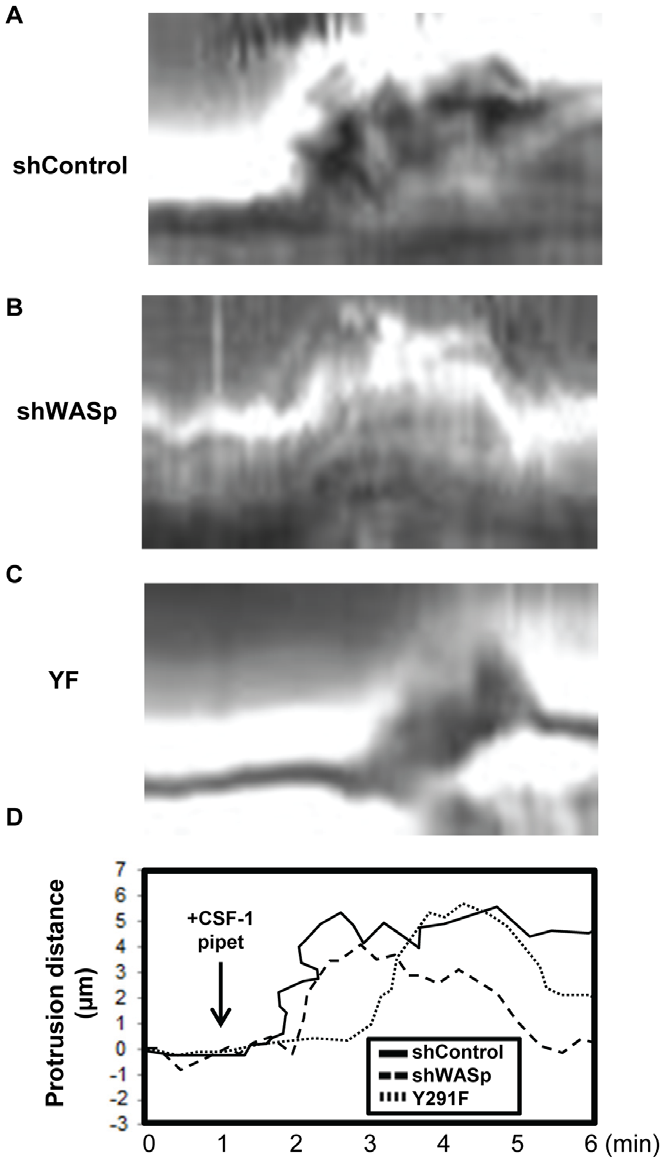
WASp and its phosphorylation are not required for directional response to CSF-1. The role of WASp in directional sensing was examined by monitoring the chemotactic responses of WASp mutant macrophages in a CSF-1 gradient. Representative kymographs from time-lapse images of (**A**) shControl, (**B**) shWASp and (**C**) Y291F cells in response to a micropipette containing CSF-1 are shown (Movies S1, S2, S3). (**D**) Overlay of the outlines of the membrane protrusions from panels **A** through **C** is shown.

**Figure 3:**
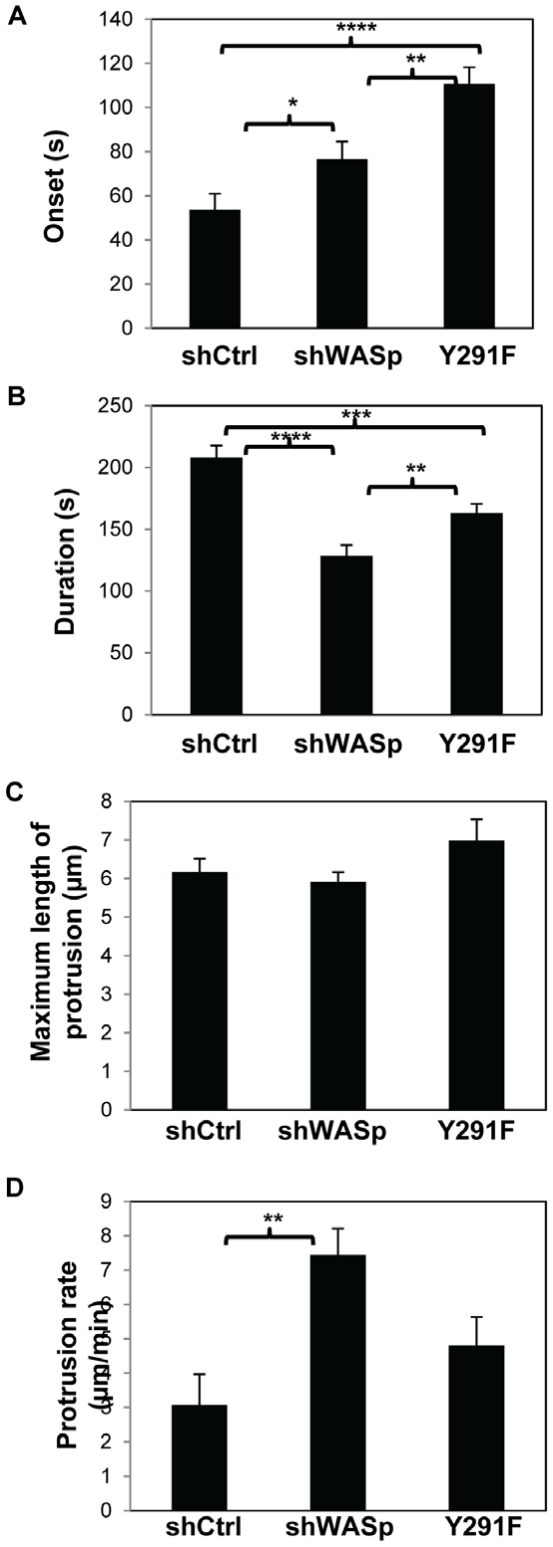
WASp and its phosphorylation are required for the persistence of chemotactic protrusion. Quantification of the protrusive responses of shControl, shWASp and Y291F cells in response to CSF-1 supplying micropipette from time lapse images are shown. (**A**) Onset of the protrusion (time after CSF-1 stimulation), (**B**) duration of the protrusion, (**C**) maximum length of the protrusion and (**D**) protrusion rate (maximum length/time it takes the protrusion to reach maximum length) in response to CSF-1 were determined. The data represent the mean of at least 30 cells from seven separate videos. Error bars represent SEM. * p<0.05, ** p<0.01, *** p<0.001 and **** p<0.0001.

Since WASp is required for directional migration of macrophages in response to CSF-1 [16] and defects in the first peak of actin polymerization in carcinoma cells is correlated with defective directional sensing [24], we proceeded to monitor the chemotactic responses of shControl, shWASp and Y291F cells in response to a directional source of CSF-1 using a micropipette. Whereas non-directional protrusions were expected to emerge from shWASp cells, analysis of the protrusions from time-lapse images showed both shControl and shWASp cells extended protrusions in a directional manner (Movies S1 and S2). Also, the directionality of shWASp protrusion was identical to that of shControl protrusions with chemotactic indices of 0.837±0.034 and 0. 834±0.045, respectively (p = 0.9568, n = 50 protrusions). Protrusions from shWASp cells, however, did not persist as long as the protrusions from shControl cells (Figure 2), and often retracted (Table 1). Detailed inspection of the time-lapse images revealed that, while there was no significant difference in the maximum length of the protrusion in the monitored time interval, the protrusions from the shWASp cells displayed delayed onset, decreased persistence and increased protrusion rate compared to shControl cells (Figure 3A–D). This result suggested that without the WASp-mediated first wave of F-actin increase chemotactic protrusions failed to persist.

**Table 1:** Summary of micropipette assay chemotactic responses.

Macrophages	Directional protrusion (%)	Retraction (%)	n (experiments; cells)
shControl	94.8±3.4	43.2±13.1	7; 49
shWASp	91.1±3.3	78.7±7.1*	7; 49
Y291F	93.8±3.2	23.3±10	10; 36
WT BMM	91.0±3.9	4.5±3.1	13; 32
WASp^−/−^ BMM	89.7±4.3	81.7±6.67***	10; 33

Percentages of cells that exhibited directional protrusions towards the micropipette and retracted within five minutes of CSF-1 stimulation are shown. Percentages were calculated by dividing the number of positive responses over the total number of cell responses recorded from each of the analyzed time-lapse images and averaging them. Indicates SEM. n indicates the total number of experiments and cells analyzed for each macrophage type.

*indicates p<0.05 compared to shControl and Y291F cells.

***indicates p<0.001 compared to wild type (WT) BMM.

Since WASp phosphorylation is important for chemotaxis the protrusion dynamics in the absence of WASP phosphorylation was examined. Phosphodeficient Y291F cells demonstrated a significant delay in the onset of the protrusions compared to shControl cells as well as to shWASp cells (Figures 2C, 3A and Movie S3). The persistence of the protrusions of Y291F cells was also reduced compared to shControl cells but was greater than that of shWASp cells. These results suggested that tyrosine phosphorylation of WASp played a complex regulatory role in the directional protrusive response of macrophages towards CSF-1.

**Figure 4:**
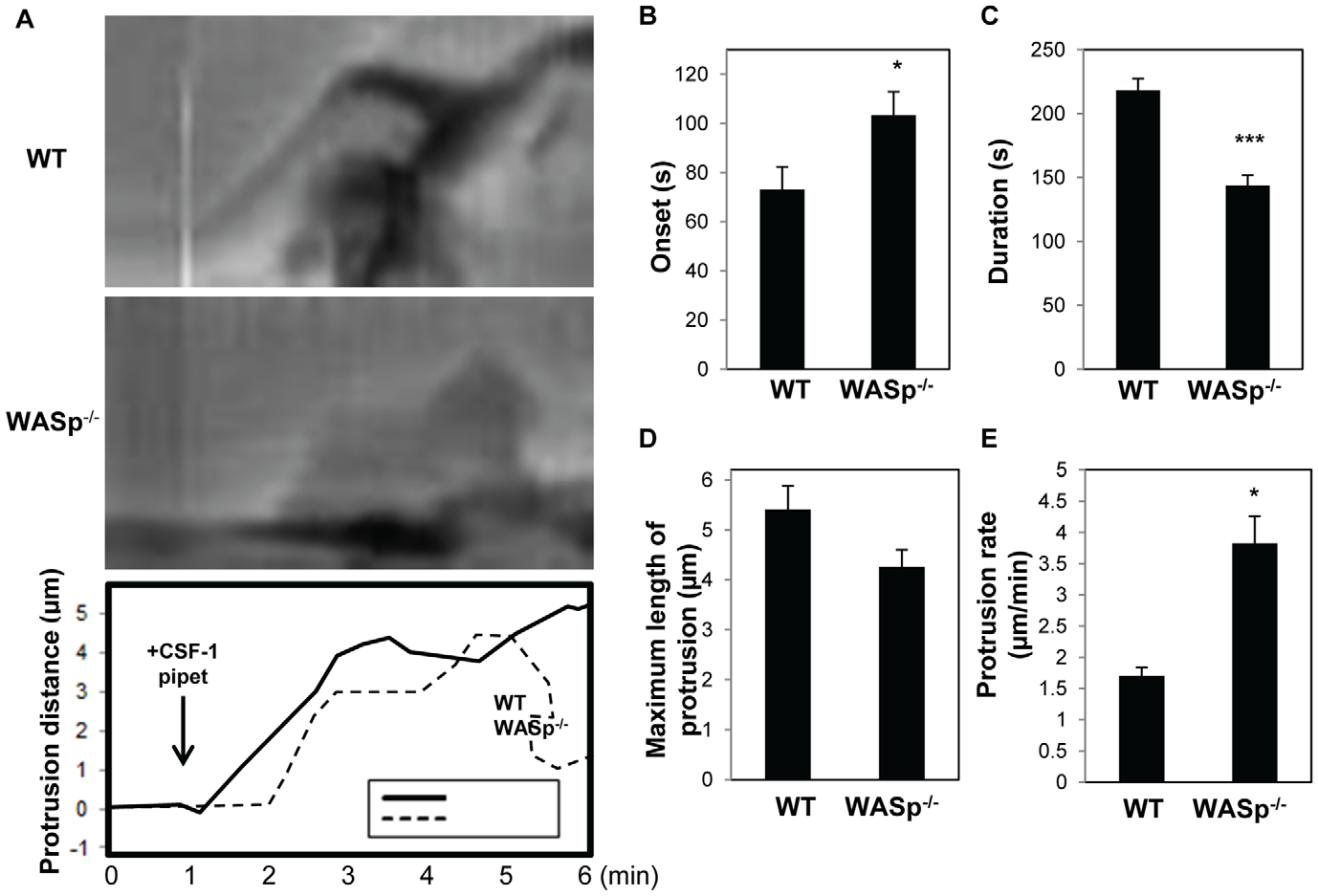
WASp is required for the persistence of chemotactic protrusion in primary BMMs. (**A**) The role of WASp in directional sensing was examined by monitoring the chemotactic responses of wild-type and WASp-deficient macrophages in a CSF-1 gradient. Representative kymographs from time lapse images of wild-type (WT, top panel) and WASp^−/−^ (middle panel) BMM in response to CSF-1 containing micropipette (Movies S4 and S5) are shown. The bottom panel shows an overlay of the outlines of the membrane protrusive activities of wild-type and WASp^−/−^ BMMs. (**B**) Onset, (**C**) duration, (**D**) maximum length and (**E**) protrusion rate (maximum length/time it takes the protrusion to reach maximum length) after CSF-1 stimulation were determined. The data represent the mean of at least 30 cells from each cell type over a minimum ten separate videos. Error bars represent SEM. * p<0.05, ***p<0.001.

To confirm our finding from the macrophage cell lines in primary macrophages, the micropipette assay was performed using bone marrow-derived macrophages (BMMs) from wild-type or WASp^−/−^ mice. In agreement with the results using cell lines, protrusions from WASp^−/−^ BMM were directional but were less persistent compared to wild-type BMM (Movies S4 and S5) and often retracted (Figure 4A and Table 1). Of note, wild-type BMMs showed lower occurrence of retraction of protrusions compared to shControl cells, yet a comparable percentage of retraction was observed between WASp^−/−^ BMM and shWASp cells. Analysis of the protrusion dynamics of WASp^−/−^ BMMs again showed delay in onset, decreased persistence and increased protrusion rate compared to wild-type BMMs (Figure 4B–D), albeit the protrusion rates of BMMs were approximately half of RAW/LR5 cells. In summary, these data demonstrate that WASp does not play a role in directional sensing but instead is required for the persistence of the protrusion when macrophages respond towards a CSF-1 gradient.

### WAVE2 localization in protrusions is unaltered in the absence of WASp

**Figure 5:**
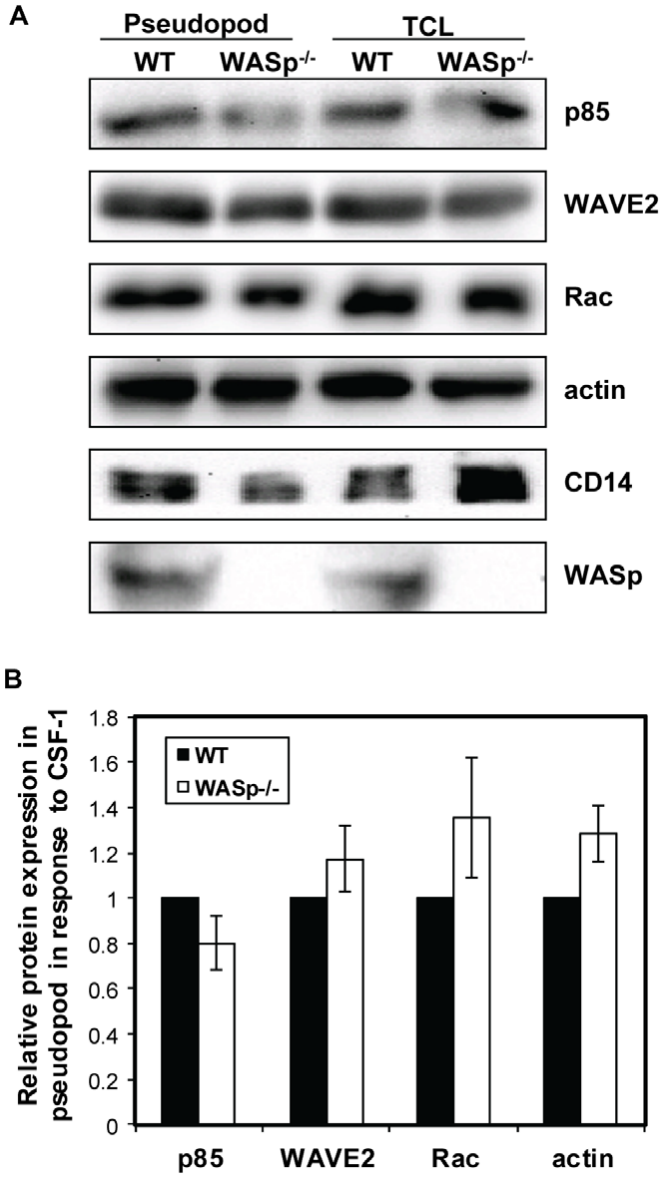
WASp is not required for the recruitment of PI 3-kinase, Rac1, WAVE2 and actin in CSF-1-elicited protrusions from primary BMMs. Chemotactic protrusions were isolated from wild-type and WASp^−/−^ BMMs in response to CSF-1 as described in Materials and Methods. (A) Representative western blots of indicated proteins from pseudopod lysates and total cell lysates are shown. (B) Protein amounts from pseudopod lysates were quantified by densitometry and normalized to the corresponding level of a non-specific membrane marker (CD14). Data is shown as relative ratio of each proteins compared to the WT BMM. n = 3 experiments. Error bars represent SEM.

Our previous work has shown that CSF-1 – elicited plasma membrane protrusions are driven by WAVE2 complex-dependent actin polymerization. We therefore examined whether WASp deficiency could affect WAVE2-mediated actin assembly, leading to altered actin dynamics. For this purpose, we isolated pseudopods from wild-type and WASp^−/−^ BMMs that were extending towards a gradient of CSF-1 (Figure 5A). Analysis of the protein contents showed that WASp did not affect the incorporation of endogenous Rac1, WAVE2, the p85 subunit of PI3K and actin in membrane protrusions (Figure 5A–B). Additionally, the relative protein levels of these proteins were similar between wild-type and WASp^−/−^ BMMs. These results are consistent with our earlier studies using RAW/LR5 macrophages where the WAVE2 complex was shown to regulate actin polymerization and membrane protrusions after five minutes of CSF-1 stimulation [14], [15], corresponding to the second wave of actin assembly. These results demonstrate that WASp, and the first wave of actin assembly in response to CSF-1, is not required for the recruitment of other proteins required for the generation of actin rich protrusion, and suggest that this is not the cause of the chemotaxis defect of WASp-deficient cells.

## Discussion

It has long been known that WASp is an important regulator of the actin cytoskeleton that is required for the proper function of immune cells, including migration of B and T cells, dendritic cells and macrophage (reviewed in [10]). However, the precise role of WASp in directed migration is unknown since WASp deficient cells still respond to chemoattractants but yet do not chemotax efficiently [16], [30]. In this study, we demonstrate that CSF-1 stimulation of macrophages results in two transient waves of actin assembly, where WASp is required for the initial peak, while WAVE2 controls the second [14], [15]. Importantly, occurrence of the second peak is independent of this first, since in the absence of WASp the second peak proceeded normally including the recruitment of PI3K, Rac1, WAVE2 and actin in directional protrusions. This first wave of actin assembly has been correlated with ability to sense the direction of a chemoattractant in carcinoma cells [24]. However, our results suggest that while WASp drives actin polymerization during the first F-actin peak, it is not required for chemotactic sensing. Rather, WASp plays a critical role in establishment of persistence of chemotactic protrusions. We also show that in the absence of WASp phosphorylation cells display a diminished first wave of actin polymerization and an accentuated defect in the initiation of the protrusions towards CSF-1. This result implicates tyrosine phosphorylation of WASp as necessary in coordinating actin cytoskeleton rearrangements needed for efficient chemotaxis.

Directional sensing is a key response that enables the cells to migrate in the correct direction. While chemoattractant receptors, such as CSF-1R, are uniformly distributed on the cell surface [31], [32], downstream components of the signaling cascade display polarized localization and/or activity [31]. Local levels of PI(3,4,5)P_3_ have been shown to determine the site of new protrusion in macrophages, whether triggered by chemoattractants or particle binding in the case of phagocytosis, which also show transient increase in actin polymerization (reviewed in [13]). In turn, temporal and spatial levels of PI(3,4,5)P_3_ are controlled by regulators such as PI3K, PLC and PTEN [24], [33], the activities of which are also regulated spatiotemporally (reviewed in [34]). PLC has been shown to be required for triggering the first but not the second wave of actin polymerization through the regulation of cofilin activity in breast cancer cells stimulated with EGF [24], [26]. PLC has also been reported to associate with CSF-1R through phosphorylation of CSF-1R tyrosine residue Y721 in a yeast two-hybrid approach using an immature myeloid cell line [35], however, a recent study using fully differentiated, immortalized BMM found that Y721, which is required for CSF-1 mediated chemotaxis, regulates CSF-1R association with PI3K but not with PLC [36]. In agreement with an important role for PI3K in macrophage chemotaxis, PI3K is required for CSF-1 induced actin polymerization and chemotaxis [19], [37] and CSF-1-elicited WASp activation in macrophages has been shown to be PI3K and Cdc42 dependent [17]. Furthermore, Cdc42 is the major activator of WASp downstream of CSF-1R activation [17] and mediates CSF-1 induced directional motility [4]. Observations similar to our findings have been reported from a study using Cdc42^−/−^ dendritic cells (DCs). Lammermann and colleagues reported that symmetry breaking and actin polymerization rates in protrusions generated in response to chemotactic cues were comparable between WT and Cdc42^−/−^ DCs. However, in the absence of Cdc42, the directional persistence was diminished and protrusions appeared to retract faster than those of WT DCs [38]. Cdc42-WASp signaling may therefore be a central regulatory mechanism for the persistence of nascent protrusions in response to chemoattractants in mononuclear phagocytes.

Several models of chemotaxis have been proposed to account for directional sensing and chemotaxis, such as those employing positive feedback loops or local excitation-global inhibition (LEGI) [34], [39]. In the positive feedback loop model, signaling molecules are selectively amplified at the anterior of the cell and thereby localize the response, leading to protrusion following directional sensing [40]. Since WASp may also act as a scaffold protein, it is plausible that WASp localizes upstream regulators, such as Cdc42 or PI3K, resulting in signal amplification required for the persistence of protrusion. In the LEGI model, receptor activation initiates a rapid and localized activating signal, followed by a slower global inhibitory signal to account for symmetry breaking and acquisition of polarized migratory morphology and, ultimately, directional migration. Xiong and colleagues recently proposed that motile cells navigate with a LEGI-biased excitable network, using simulations based on a model that accounts for the two transient waves of signaling events and excitability of the biochemical network that is activated within the first five minutes of chemoattractant stimulation of migrating cells [41]. These simulations can predict cellular responses under directional chemoattractant stimulatory conditions where the parameters of the LEGI-biased excitable network model can be altered to mimic known motility defects. For example, by lowering the excitability of the network parameter, such as decreasing the threshold of a positive feedback loop, this LEGI-model predicted that the spontaneous activity of the system would be eliminated but directional sensing would be maintained. Furthermore, Xiong and colleagues drew attention to how this perturbance in the excitable network is reminiscent of cells treated with Latrunculin A, which prevents actin polymerization, similar to WASp-deficiency. Indeed, *Dictyostelium* cells with reduced WASp expression [42] have reduced F-actin levels following cAMP stimulation, and fewer and mislocalized barbed ends, indicative of the role of WASp in actin polymerization during chemotaxis. However, whereas F-actin could not distribute towards the direction of the cAMP gradient, Akt was able to localize correctly to the plasma membrane, indicating that the pathways regulating PI(3,4,5)P3_3_ levels remained intact. Our results are consistent with the LEGI-biased excitable model since WASp-deficient macrophages respond to directional CSF-1 stimulation but the protrusions were not maintained.

The role of phosphorylation of tyrosine residue 291 of WASp has been studied in detail *in vitro* [29], [43]. While the phospho-deficient WASp showed comparable rates of actin polymerization *in vitro* [44], our *in vivo* characterization revealed that actin polymerization by Y291F cells in response to CSF-1 was identical to shWASp cells, consistent with the inability of Y291F WASp expressing cells to migrate towards CSF-1 [18]. Interestingly, Y291F showed an even greater defect in the initiation of the chemotactic response compared to shWASp cells in the micropipette assay, suggesting that tyrosine phosphorylation of WASp does not appear to simply regulate the efficiency of actin polymerization. Instead, the difference in the cellular responses of Y291F cells to control cells during upshift and directional CSF-1 stimulations may be due to mistargeting of WASp during chemotactic responses, since WASp phosphorylation has been suggested to play a role in the subcellular localization of active WASp in macrophages [18]. Taken together, these results suggest that localization of WASp by tyrosine phosphorylation may be required to restrict actin polymerization towards the chemoattractant, consistent, at least in part, with the LEGI model.

Alternatively, WASp may play a role in adhesion of chemotactic protrusions leading to their maintenance since protrusions will often retract if not stabilized by substrate adhesion even in the continued presence of a chemoattractant gradient, as reported in *Dictyostelium* amoebae, carcinoma cells and neutrophils [45], [46], [47]. It is well characterized that monocyte-derived cells employ F-actin-rich structures called focal complexes and podosomes to adhere and migrate [48], [49]. Importantly, WASp is required for the formation of podosomes [50] and tyrosine phosphorylation of WASp has been shown to regulate actin dynamics inside podosomes [18]. Therefore it is possible that WASp may be required to stabilize the chemotactic protrusions by promoting cell-substrate interactions. While lack of adhesion may account for the increased retraction rate observed in the absence of WASp, it does not account for the observed delay in the onset of protrusions in the absence of WASp or its phosphorylation (Figure 3) suggesting that WASp may play multiple roles in chemotactic protrusions.

In conclusion, our data show that WASp is required for the first wave of actin assembly in response to CSF-1 that leads to the establishment of persistence of chemotactic protrusions of macrophages. We also show that tyrosine phosphorylation state of WASp plays a role in the initiation of chemotactic protrusion. A key question remains, which is how WASp activity is regulated and maintained in a temporally and spatially controlled manner for successful protrusion persistence and chemotaxis to take place. While further studies will be required to elucidate the molecular mechanism that regulate WASp activity during macrophage migration, the data presented herein highlight the critical role of WASp in the establishment of persistence of protrusion in macrophage chemotaxis.

## Supporting Information

Movie S1 **Time lapse of shControl RAW/LR5 cells responding to a directional source of CSF-1.** The movie corresponds to Figure 2. The movie plays at 7 frames per second.
(MOV)

Movie S2 **Time lapse of shWASp RAW/LR5 cells responding to a directional source of CSF-1.** The movie corresponds to Figure 2. The movie plays at 7 frames per second.
(MOV)

Movie S3 **Time lapse of Y291F RAW/LR5 cells responding to a directional source of CSF-1.** The movie corresponds to Figure 2. The movie plays at 7 frames per second.
(MOV)

Movie S4 **Time lapse of WT BMMs responding to a directional source of CSF-1.** The movie corresponds to Figure 4. The movie plays at 7 frames per second.
(MOV)

Movie S5 **Time lapse of WASp^−/−^ BMMs responding to a directional source of CSF-1.** The movie corresponds to Figure 4. The movie plays at 7 frames per second.
(MOV)
